# A Patient-Derived Organoid Platform from TUR-P Samples Enables Precision Drug Screening in Advanced Prostate Cancer

**DOI:** 10.3390/cancers17243973

**Published:** 2025-12-12

**Authors:** Zaukir Bostan Ali, Mooktapa Plikomol, Tanan Bejrananda, Paramee Thongsuksai, Pokphon Khirilak, Natthapon Khongcharoen, Karan Ulhaka, Ratsamaporn Nontikarn, Onpawee Phanthuvet, Pasarat Khongkow

**Affiliations:** 1 Department of Biomedical Sciences and Biomedical Engineering, Faculty of Medicine, Prince of Songkla University, Songkhla 90110, Thailand; zakbostan@gmail.com (Z.B.A.); mooktapa.mp@gmail.com (M.P.);; 2 Urology Unit, Department of Surgery, Faculty of Medicine, Prince of Songkla University, Songkhla 90110, Thailand; t13ers@hotmail.com; 3 Department of Pathology, Faculty of Medicine, Prince of Songkla University, Songkhla 90110, Thailand; 4 Translational Medicine Research Center, Faculty of Medicine, Prince of Songkla University, Songkhla 90110, Thailand

**Keywords:** advanced prostate cancer, patient derived organoids (PDOs), transurethral resection of prostate (TUR-P), drug screening

## Abstract

**Background:** Patient-derived advanced prostate cancer organoids have been developed to mimic tumor heterogeneity and beneficially predict optimized drugs for specific patients. The organoids are promising functional drug screening models which can capture patient outcomes. However, organoid development from transurethral resection of the prostate (TUR-P) has been hampered by a low success rate, and the cost of culture should be reduced for realistically clinical settings. In our study, we aimed to improve the success rate and reduce the cost of establishing advanced prostate cancer organoids from TUR-P specimens. **Methods:** We optimized and improved both the organoid culture protocol and the fetal bovine serum (FBS) based-organoid culture medium, which is suitable for performing drug testing in a short turnaround time. To confirm that the generated organoids could recapitulate the tumor heterogeneity of original tissues, the organoids were validated with histological, immunohistochemical, and genomic analyses. **Results:** Following the optimized protocol, we successfully generated organoids in approximately 18 out of 29 cases (or 62.07%), which exhibited effective growth and survival. In addition, we found that the established organoids efficiently identified and captured tumor characteristics present in their matched original tissues, as indicated by histological, immunohistochemical, and comprehensive genomic analysis. As a proof of concept for personalized medicine, the generated organoids were treated with anti-cancer drugs, including docetaxel and enzalutamide in parallel with the clinical treatments. Interestingly, the in vitro drug screening results were positively correlated with the patient outcomes at the clinical level. **Conclusions:** Taken together, the established APC organoids were able to precisely predict patients’ outcomes for treatment decision-making within a month in a cost-effective manner.

## Simple Summary

Advanced prostate cancer (APC) is highly heterogeneous, often leading to treatment resistance. In this study, we optimized a cost-effective protocol to establish patient-derived organoids (PDOs) from transurethral resection of the prostate (TUR-P) samples, achieving a 62.07% success rate. These organoids preserved key features of the original tumors and enabled precise drug screening. Notably, in vitro responses to drugs like docetaxel correlated with clinical outcomes, demonstrating strong potential for guiding personalized treatment decisions in APC patients within a clinically relevant timeframe.

## 1. Introduction

Prostate cancer is the second most diagnosed cancer among males worldwide. Over the past decade, the percentage of patients with the disease has increased from 3.9% to 8.2%, especially at an advanced stage [1]. Advanced prostate cancer is a highly heterogeneous tumor that cannot be cured with surgery or radiation alone. Systemic therapy is needed to control the disease [2]. Although systemic therapy can improve the overall survival rate of patients [3], the wide variability in patient outcomes is critically challenging. Thus, several approaches based on precision medicine concepts are being developed to precisely predict patient outcomes and improve treatment recommendations. The main aim is to tailor clinical management based on patient information. Recently, the cancer genome atlas of prostate cancer patients has been analyzed and reported as a comprehensive molecular portrait. This is beneficial for discovering novel targeted agents for patients, followed by genomic profiling or targeted therapies. For example, advanced prostate cancer patients who have the BRCA mutation can be alternatively treated with FDA-approved Olaparib for advent treatment [4,5,6]. Despite the promises of targeted therapies, some cancer patients could eventually develop resistance to them. This is associated with a change in the set of targeted genes or stimulation of tumor growth via a different pathway. In addition, most targeted therapies are limited to evaluation in clinical trials and can only predict clinical outcomes, lacking functional validation in an individual patient [4].

Based on this limitation, in vitro functional drug screening models have been developed to precisely predict and validate cancer patient outcomes. The models have emerged to mimic tumor characterization and their microenvironment. Initially, two-dimensional culture models provided less time-consuming and simplified methods for initiating drug screening. However, this system failed to capture the systemic nature of tumors, leading to weak predictive ability [7,8]. To preserve tumor heterogeneity, patient-derived xenograft models have been generated by transplantation of carcinoma cells into animal models. These models closely resemble in vivo tumor biology and their microenvironment, but they consume a large amount of time for drug testing [9,10]. Hence, biological models which are more reliable and feasible for drug screening platforms are urgently needed for precise prediction of cancer patient outcomes.

In recent years, three-dimensional culture models from prostate cancer patient-derived tissues, also known as “organoid models”, have been developed to provide real tumor settings and perform drug screening. Previous studies reported that prostate cancer organoids, as original tissues, are promising for the capture of tumor heterogeneity at both cellular and genetic levels. Moreover, the organoids are efficient in performing functional drug screening tests [11,12,13,14,15,16]. Prostate cancer organoids can be generated from several collective types of specimens, such as transurethral resection of the prostate (TUR-P) and radical prostatectomy (RP). Although TUR-P is less invasive, the success rate of establishing organoids has been relatively low, approximately 28.57% [11,17]. The specimens are exposed to electric wires during surgery, leading to damage of some cell populations [2]. Apart from tissue collection being associated with the success of organoid derivation, the culture medium is also important to support organoid growth. Based on conventional organoid culture media [15,18], a lot of studies have improved the method to minimize components and reduce the cost of culture [19,20,21].

In this study, we improved the culture protocol for establishing advanced prostate cancer organoids from tissue resections, especially TUR-P samples. We optimized important procedures and minimized the fetal bovine serum (FBS)-based organoid culture medium to effectively support short-term experiments. We found that the generated organoids could preserve the tumor heterogeneity of the matched original tissues. To prove the precision medicine concept, the generated organoids were used for in vitro drug testing in parallel with clinical treatments. The organoids could recapitulate patient outcomes and possibly be used as promising functional drug screening models.

## 2. Materials and Methods

### 2.1. Case Selection and Obtainment of Informed Consent

The study protocol was reviewed and approved by the Human Research Ethics Committee of the Faculty of Medicine, Prince of Songkla University (REC.64-565-25-2). Patient-derived cancer organoid handling, culture, and validation protocols were approved by the Office of Human Research Ethics Committee (HREC), Faculty of Medicine, Prince of Songkla University. All participating patients in this study were asked to voluntarily sign informed consent forms which were approved by the committee. The patients could withdraw their consent at any time, whereupon their tissues would be promptly removed. The inclusion criteria for the collection of the 29 prostate patient samples were as follows: the patients were diagnosed with advanced prostate cancer and had a Gleason score greater than or equal to 6; they needed surgery in one part of the treatment plan; and they had never been treated with any anti-cancer drugs before.

### 2.2. Patient-Derived Specimen Collection and Handling

Surgically resected tissues of advanced prostate cancer were obtained from Songklanagarind Hospital. The tissues were stored in working medium, composed of DMEM/F12 medium (1:1; cat. no.11995-065 and 11765-054), Penicillin/streptomycin (1% *v/v*; Gibco 15140-122), HEPES (1% *v/v*; Gibco 15630-080), and Glutamax (1% *v/v*; Gibco 35050-061), on ice (4 °C) until the start of the experiment. These medium components were purchased from Gibco (Thermo Fisher Scientific, Waltham, MA, USA). The laboratory process must be conducted in a biosafety cabinet using Biosafety Level 2 techniques. Upon arrival, the biospecimens were transferred into a cell culture Petri dish, and then they were washed with cold PBS and cold working medium. The tissues were cut into 1–2 mm^3^ pieces. Some tissues were aliquoted into cryovial tubes and snap-frozen at −80 °C for DNA analysis. Some of them were fixed with neutral buffered formalin solution (10%; HT501128, Sigma Aldrich, Singapore) for histopathological and immunohistochemical staining. A sufficient number of tissues were set aside for mincing further to generate organoids.

### 2.3. Establishing Organoids from Advanced Prostate Cancer Specimens

The remaining tissues were minced with surgical scissors into 0.5–1 mm^3^ pieces, and then the minced tissue (approximately 40 mm^3^) was digested in 1 mL of digestion medium containing working medium supplemented with collagenase type II (5 mg/mL; Cat No. 17101-015, Gibco, Thermo Fisher Scientific, Waltham, MA, USA) for 15 min at 37 °C on a shaking platform. The complete digestion was assessed under microscope, and the reaction was terminated by adding 500 μL of FBS-based organoid culture medium, containing DMEM/F12 medium (1:1; 11995-065 and 11765-054), Penicillin/streptomycin (1% *v/v*; 15140-122), HEPES (1% *v/v*; Cat No. 15630-080), and Glutamax (1% *v/v*; Cat No. 35050-061) and supplemented with FBS (10%; Cat No. 10270-106). These medium components and the FBS supplement were purchased from Gibco (Thermo Fisher Scientific, MA, USA). After that, the material was shaken vigorously by pipetting and filtered with a 100 μm strainer. The filtrate was collected and centrifuged at 500× *g* in 4 °C for 5 min. The pellet was resuspended in cold growth factor-reduced Matrigel (10 mg/mL; Cat No. 356231, Corning, Steuben County, NY, USA). The gel suspension was seeded at about 40 μL per drop on a pre-warmed 24-microwell tissue culture plate. Then, the drops were incubated at 37 °C for 20 min to solidify. Once the Matrigel became stable, 500 μL of FBS-based organoid culture medium was gently added into the wells, and the plate was transferred back to the incubator at 37 °C with 5% CO_2_ for organoid culturing. The organoids were visualized and captured with a BioTek Lionheart FX automated microscope (Agilent, Santa Clara, CA, USA) and then analyzed using licensed GEN5.0 software (Agilent, Santa Clara, CA, USA) and open-access Image J software (version 2.14.0/1.54f) to quantify the average diameter and area of the individual organoids. After culturing for 5 days, the percentage of growth rate was calculated using an equation [22,23]:

$$\%\;organoid\;growth\;at\;day\;5=\;\left[\frac{Area\;of\;organoid\;at\;day\;5-\;Area\;of\;organoid\;at\;day\;1}{Area\;of\;organoid\;at\;day\;1}\right]\times 100$$

### 2.4. Histology and Immunohistochemical Analysis

Organoids were harvested and fixed with neutral buffered formalin solution at room temperature for 30 min (10%; Sigma Aldrich; HT501128). Then, they were collected and centrifuged at 500× *g* for 5 min. The fixed organoids were suspended in 2% of agarose, followed by the AMeX method, together with the matched tissues. The paraffin boxes containing organoids and tissues were cut with a microtome and stained with hematoxylin & eosin (H&E) and specific biomarkers, including Cytokeratin type 5/6 (1:500; Cat No. M7237, Dako, Agilent, Santa Clara, CA, USA), Cytokeratin type 8 (1: 800; Cat No. PA5-29607, Invitrogen, Thermo Fisher Scientific, Waltham, MA, USA), and Androgen receptor (1:500; Cat No. 5153S, Cell signaling, Danvers, MA, USA) biomarkers.

### 2.5. Short Tandem Repeat (STR) and Digital Droplet PCR

To indicate that the original patient tumors and organoids had identical characteristics, short tandem repeat analysis was performed at 25 loci on different chromosomes (including Y Indel, D8S1179, D21S11, D7S820, CSF1PO, D3S1358, TH01, D13S317, D16S539, D2S1338, D19S433, vWA, TPOX, D18S51, D5S818, FGA, Amelogenin, Penta D, D22S1045, Penta E, D10S1248, D12S391, D1S1656, D2S441, and D6S1043). Genomic samples were amplified using the VeriFiler™Plus PCR Amplification Kit (A35495, Thermo Fisher Scientific, Waltham, MA, USA). They were run through the 3500 Genetic Analyzer for human identification (Cat.No. 4406017, Thermo Fisher Scientific, Waltham, MA, USA) and reported in GeneMapper software version 1.6 (Cat No. 4475073, Thermo Fisher Scientific, Waltham, MA, USA). To confirm that the PDOs could maintain cancer-associated genes as in the original tissue, the BRCA1 copy number assay (Assay ID: dHsaCP2500367) was performed using Digital droplet PCR, following the manufacturer’s instructions (Biorad, Hercules, CA, USA). Then, the QuantaSoft software version 1.7.4.0917 (Biorad, Hercules, CA, USA) was used to measure the number of droplets that were negative and positive for HEX and FAM fluorophores in a sample. The positive results were fitted with a Poisson distribution to determine the absolute initial copy number of BRCA1.

### 2.6. Whole-Exome Sequencing for the Genetic Profile Comparison of Tissue and Organoids

The DNA extracted from the patients was sequenced using the NovaSeq 6000 (Illumina, San Diego, CA, USA). The library preparation capturing regions was performed using the Agilent SureSelect Human All Exon v8 kit to produce 2 × 150 bp reads (Agilent, Santa Clara, CA, USA. First, Fastp was performed to check the quality of the WES data. The passed WES data were analyzed using the GATK best-practice workflows developed by the Broad Institute. The quality-passed reads were aligned with the reference genome GRCh38 using BWA-MEM (version 2). Then the alignment sequences were processed with MarkDuplicatesSpark for marking PCR duplicates and sorting, resulting in a sorted BAM file. For recalibration of the data, GATK’s BaseRecalibrator and ApplyBQSR tools were used to recalibrate the sorted BAM file. The GATK Mutect2 was then used to generate a VCF for identifying the somatic short variants (SNVs + Indels). The GATK somatic pipeline was based on the best-practice workflow for somatic mutation detection established by the Broad Institute in tumor-only mode (https://gatk.broadinstitute.org/hc/en-us/articles/360037593851-Mutect2 (accessed on 2 December 2024)). The variants that had a depth coverage of 10× and only those filtered as “PASS” were used for variant annotation. Those variants were annotated by ANNOVAR (version 20200607) [24]. The 87 oncogenes that most commonly occur in advanced prostate cancer were chosen to compare variants and mutation profiles. The similarity of tumors and organoids was compared with Venn diagrams using python.

### 2.7. In Vitro Drug Screening

For drug screening, organoids were first dissociated into smaller organoid fragments by vigorous pipetting. The resulting suspension was passed through 70 µm and 100 µm cell strainers to remove large aggregates and single cells. Organoid fragments within the 70–100 µm size range were collected, as this size range provides consistent morphology and growth characteristics. The size-selected organoids were pelleted by gentle centrifugation and resuspended in cold Matrigel (10 mg/mL; Cat no. 356231, Corning, Steuben County, NY, USA). Approximately 10–30 organoids were seeded per well by counting the particle density under the microscope. For each well, ~10 µL of the organoid–Matrigel mixture containing the defined number of organoids was dispensed into a pre-warmed 96-well tissue culture plate. After Matrigel polymerization at 37 °C, 100 µL of pre-warmed prostate cancer organoid medium was gently added to each well, and the plate was returned to the incubator. After 24h of organoid culture, the culture medium was replaced with culture medium containing Docetaxel (0, 0.1, 1, 10, and 100 nM) and Enzalutamide (0, 0.01, 0.1, 1, and 10 μM). At day 6 of treatment, the culture medium with or without the drug was removed, and Calcein AM and BOBO-3 iodide reagent were added and mixed to analyze cell viability following the guidelines of the LIVE/DEAD Viability/Cytotoxicity Kit (R37601, Thermo Fisher Scientific, Waltham, MA, USA). Then, the stained organoids were captured and analyzed with the BioTek Lionheart FX automated microscope (Agilent, Santa Clara, CA, USA) and licensed GEN5.0 software (Agilent, Santa Clara, CA, USA), respectively. In addition, to measure ATP in living cells, the treated organoids were assessed using CellTiter-Glo^®^ Measures ATP (Cat.No. G7572, Promega, Madison, WI, USA).

### 2.8. Statistics

Data analysis was performed, and graphs were created using Excel and Graphpad software (version 10.4.2). Drug screening was performed at least in duplicate. Quantitative variable results were presented as the means ± SDs, and *p* values were calculated using Welch’s unpaired *t*-test. The correlation between in vitro drug screening and patient response was calculated using *p* values, sensitivity, and specificity by Fisher’s exact test [25,26].

## 3. Results

### 3.1. Improving the Protocol for Establishing Advanced Prostate Cancer Organoids

**Figure 1:**
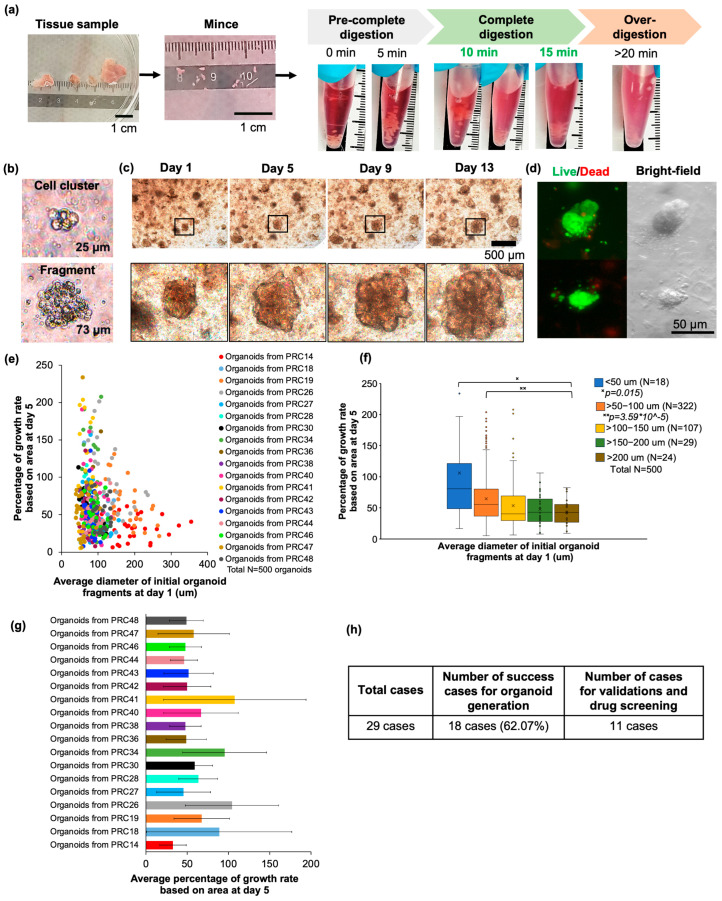
Establishing advanced prostate cancer organoids. (**a**) Schematic diagram depicting the critical steps and optimized digestion conditions which were used for organoid generation. (**b**) Organoid fragments and cell clusters after complete digestion. (**c**) Color bright-field images showing the growth of prostate cancer organoids at days 1, 5, 9, and 13. (**d**) Bright-field and fluorescent images of the prostate cancer organoids at day 5 presenting the viability of organoids (green: live cells; red: dead cells). (**e**) Dot plot in which each dot represents an individual organoid fragment, with dot colors categorized by case number. and (**f**) box-and-whisker plot indicating that the initial diameters of isolated organoids could affect the percentage growth rates of organoids (N = 500 analyzed organoids from 18 cases). (**g**) Five-day average of percentage growth rates based on area from 18 patient-derived organoid lines (means ± SDs from an average of 30 analyzed organoids in each case). (**h**) Summary of the successful cases of generated organoids and the number of cases for validation and drug testing.

Advanced prostate cancer organoids were generated from resections of TUR-P (N = 28 cases) and RP (N = 1 case) with distinct clinical and pathogenic characterizations, as detailed in Table 1. We succeeded in generating advanced prostate cancer organoids in about 18 out of 29 cases (or 62.07%), which exhibited a growth rate greater than 30% based on area expansion after 7 days of culture (Figure 1). To improve the success rate, we optimized a culture protocol for generating organoids from cell clusters and tissue fragments. This protocol was beneficial in reducing both the time and cost for organoid generation and supporting organoid growth by cell–cell and cell–stroma interaction. In this critical step, we firstly optimized enzymatic digestion conditions, especially incubation time, to maximize organoid fragmentation and cell cluster derivation. The different time points were observed at 0, 5, 10, 15, and 20 min. Initially, the tissue fragments exhibited a dense structure, and the suspension appeared clear. Upon complete digestion (approximately 10 to 15 min after digestion), the tissue fragments and suspension were soft and intermediately cloudy, but the tissue pieces were still visible inside the tube. When sampling the suspension under a microscope, the majority of organoid fragments and cell clusters less than 100 µm in average diameter could be observed. In contrast, if the digest condition time was about 20 min or longer, the organoid fragments and cell clusters were rarely observed because of over-digestion (Figure 1a,b).

Next, to identify the optimal size range of initial tissue fragments that promotes the most effective organoid growth, we categorized the fragments into five groups: <50 µm, 50–100 µm, 100–150 µm, 150–200 µm, and >200 µm. The growth performance of each group was subsequently evaluated. The average growth rates were 105.93% (N = 18), 64.52% (N = 322), 53.30% (N = 107), 47.31% (N = 29), and 42.73% (N = 24), respectively. These results indicate that the initial fragment size markedly influences organoid proliferation efficiency. Notably, tissue fragments with diameters less than 100 µm exhibited significantly enhanced growth rates after 5 days of culture. Therefore, for subsequent experiments, we cultured organoids using initial tissue fragments smaller than 100 μm to ensure optimal growth capacity (Figure 1e,f).

In parallel, we also investigated whether our optimized FBS-based organoid culture medium could efficiently support growing organoids and maintain survival capacity. The results demonstrated that advanced prostate cancer organoids exhibited continuous size expansion, with the average growth area increasing from 32.62% to 107.52%. To assess viability, organoids were stained with Calcein AM (live-cell indicator) and BOBO-3 Iodide (dead-cell indicator). Fluorescence imaging revealed that the majority of organoid areas were composed of viable cells, confirming robust survival under this culture condition (Figure 1c,d,g). In addition, to demonstrate the capacity of FBS-based medium for organoid maintenance, the mature organoids underwent a sub-culture process. The criteria for re-growing organoids were a diameter greater than 200 μm, high density (or sign of a dark center), and Matrigel qualification. According to the results, the organoids were successfully regenerated within a couple of weeks, and a predominant number of cases had the capacity of maintenance over two passages (Supplementary Figure S1). Taken together, the improved culture procedure and FBS-based medium were the critical keys in enhancing the success rate of TURP-derived organoid generation. Despite these advances, some cases failed to proliferate, primarily due to prolonged ischemia time, microbial contamination, or inadequate epithelial content, all of which reduced viability and prevented sustained organoid growth.

### 3.2. Validation of Advanced Prostate Cancer Organoids Compared with Original Tumors

**Figure 2:**
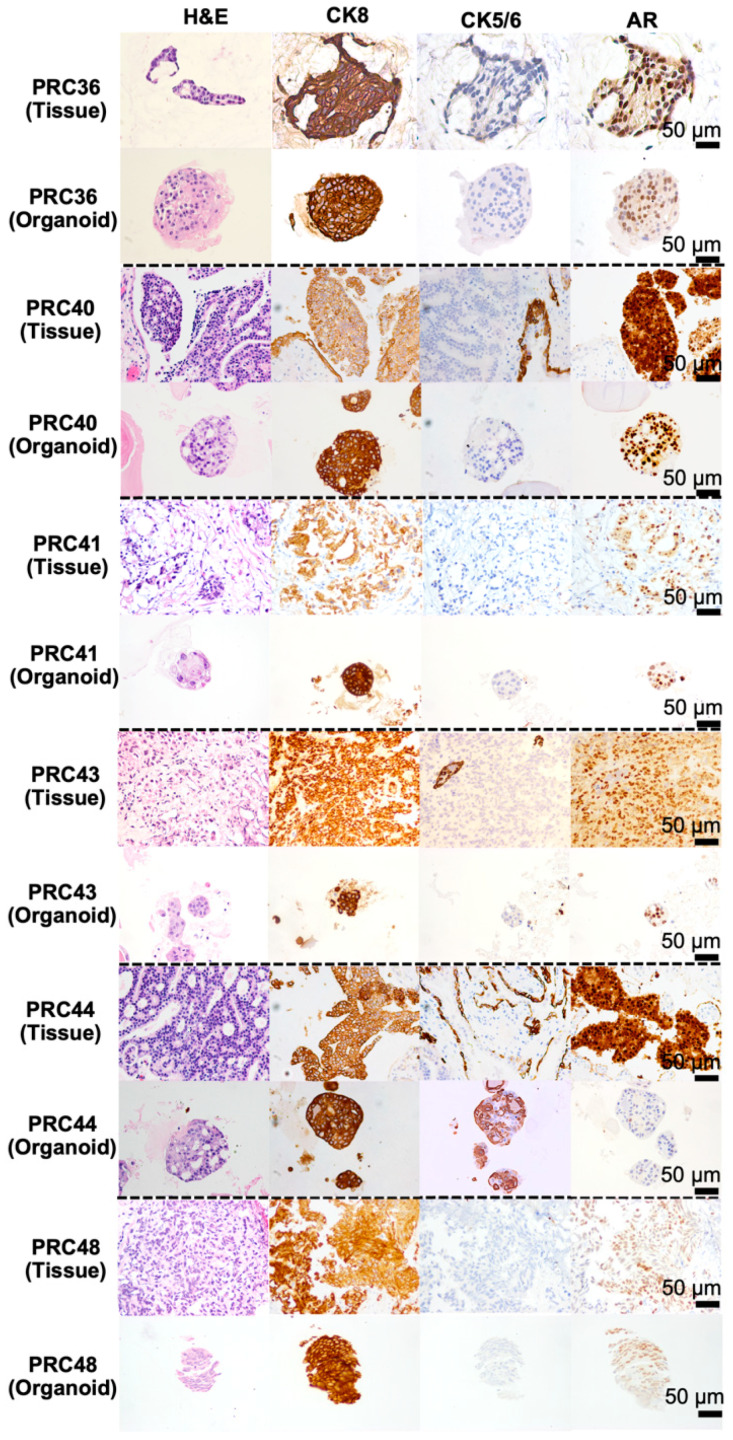
Representative H&E and immunohistochemical staining of organoids and their matched tumors. Hematoxylin and eosin (H&E) staining and immunohistochemical analyses for Cytokeratin 8 (CK8), Cytokeratin 5/6 (CK5/6), and Androgen Receptor (AR) are shown for each patient case to demonstrate the preservation of key prostate cancer epithelial markers within each matched tumor–organoid pair.

After successfully generating the organoids, we next confirmed that the organoid–tissue pairs preserved the tumor heterogeneity of the original samples. This validation was performed through histological characterization, immunohistochemical profiling, and genomic analysis. Hematoxylin and eosin (H&E) staining revealed that the organoids were predominantly composed of cancerous epithelial cells, whereas the original tissues displayed epithelial cell rearrangements surrounded by connective tissue components. The generated organoids typically exhibited a dense structural morphology; however, in some cases, both dense and cystic formations were observed within the same culture batch. Morphologically, the organoids recapitulated typical malignant features, including enlarged and polymorphic nuclei and pleomorphic cell shapes. Furthermore, prostate cancer organoids and their corresponding tissues were analyzed by immunohistochemical profiling of Cytokeratin 5/6 (CK5/6), Cytokeratin 8 (CK8), and Androgen Receptor (AR). CK5/6 and CK8 are commonly used markers to distinguish basal and luminal epithelial cells, respectively. In advanced prostate cancer, strong CK8 expression with weak or absent CK5/6 expression is typically observed, whereas AR—a hormone receptor—remains highly expressed and is characteristic of prostatic adenocarcinomas. Consistent with these patterns, both the organoids and their corresponding tissues showed negative to weakly positive CK5/6 expression, strong CK8 positivity in all cases, and positive AR expression with comparable levels. These findings confirm that the generated organoids originated from prostate cancer cells and faithfully retained the characteristics of advanced-stage disease, consistent with their tissue origins (Figure 2 and Figure S2).

**Figure 3:**
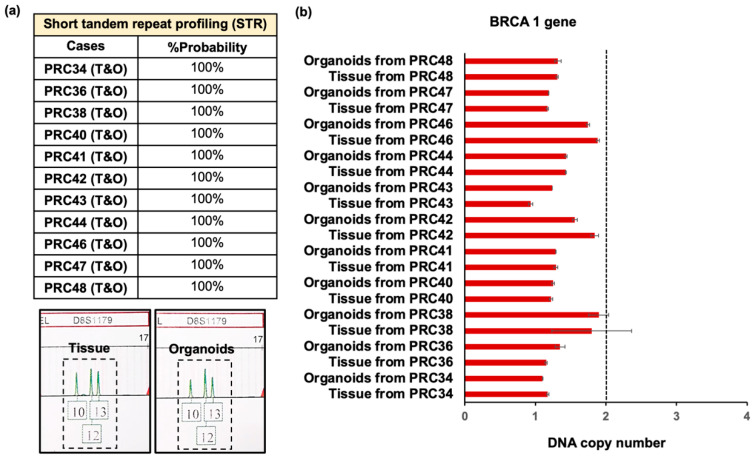
Genomic analyses of tissue–organoid pairs. (**a**) Short tandem repeat analysis (STR) of 25 loci in different chromosomes indicated that original tissue and organoids had identical characteristics, with 100% probability (T = original tissue; O = organoids). Additionally, one pair preserved the type 2 tri-allelic pattern. (**b**) Bar chart showing the BRCA1 gene, highlighted by red coloring, showing conserved copy number variation (CNV) losses among organoid–original tissue pairs (N = 11 analyzed cases and at least triplicate experiments in each case).

In addition, to validate that the tissue–organoid pairs could be identified at the tissue level, we also analyzed the similarity of the organoids and matched tissues using a short tandem repeat (STR) analysis of 25 loci in different chromosomes. The STR profiling reported that the tissue–organoid pairs could be identified with about 100% probability. Interestingly, one of the generated organoids maintained the genetic feature of the type 2 tri-allelic pattern [27], similar to the original tumor (Figure 3a). Moreover, digital droplet PCR (ddPCR) analysis was performed to assess *BRCA1* copy number variation (CNV) in both the original tumor tissues and their matched organoids. Quantitative ddPCR measurements demonstrated that all samples exhibited *BRCA1* copy numbers below the expected diploid value (approximately two copies), consistent with CNV loss as defined in previous studies [28,29,30] (Figure 3b). Across all cases, *BRCA1* copy numbers were reduced to approximately 1.0–1.8 copies, indicating a heterozygous deletion, whereas no sample showed a nondetectable reference signal suggestive of homozygous loss. Notably, the *BRCA1* CNV profiles of the organoids closely matched those of their corresponding tumor tissues with 100% concordance, confirming that the PDOs faithfully preserved the genomic alteration patterns of their parental tumors.

**Figure 4:**
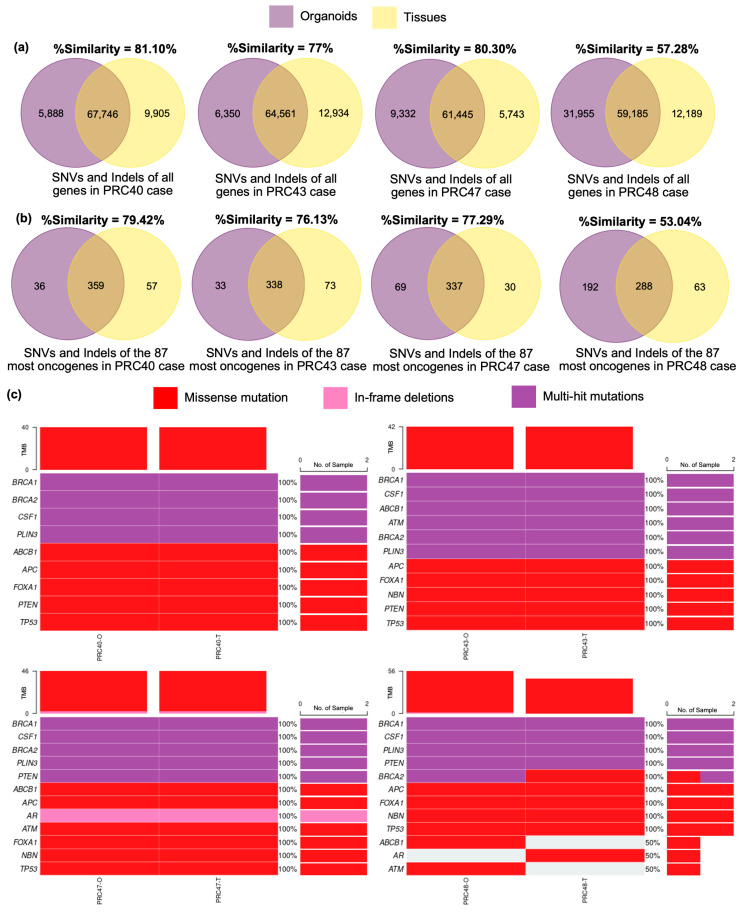
Whole-exome sequencing analysis of organoids compared with original tissues. Venn diagram of (**a**) single-nucleotide variants (SNVs) and small insertions and deletions (Indels) of all genes depicting the similarity of organoid–tissue pairs (N = 4 analyzed cases). (**b**) Venn diagram of SNVs and small Indels of the 87 most prostate cancer-associated genes showing the similarity of organoids and original tissues. (**c**) Heatmap showing somatic mutations of the 87 most prostate cancer-associated genes, including missense mutations, in-frame deletions, and multi-hit mutations.

Furthermore, we employed whole-exome sequencing to characterize and confirm that organoids and tissues could preserve the gene mutation profiles of original tissue. The PRC40, -43, and -47 cases were obtained from castration-sensitive prostate cancer (CSPC) patients, and PRC48 was derived from a castration-resistant prostate cancer (CRPC) patient. The results demonstrated that organoid and tissue pairs had a similarity in the range of 57.28% to 81.10% (or, on average, 73.92%) for single-nucleotide variants (SNVs) and small insertions and deletions (Indels) of all genes. We also compared the organoids and tissues with somatic SNVs and Indel mutations of the 87 most prostate cancer-associated genes [13,31,32]. The organoid and tissue pairs could preserve the mutations of the gene lists with 53.04% to 79.42% similarity (or, on average, 71.47%) (Figure 4a,b).

A correlation heatmap demonstrated that the organoids could mostly preserve the most relevant advanced prostate cancer genes. For example, *FOXA1*, a transcription factor and AR-mediated gene [33], was found in all cases and had been totally preserved in tissue–organoid pairs with missense mutations. *TP53*, *APC*, and *PTEN* are the tumor suppressor genes most commonly observed in advanced cancer states [34]. The results showed that tissues and organoids in all cases displayed mutations in these genes that matched. In addition, *AR* mutations, relating to progression to CRPC [31,35], were observed in one out of three CSPC cases. PRC47 showed an in-frame deletion. Although the PRC48 CRPC case was detected, we found this in the tissue only. We also observed the genes associated with DNA repair and aggressive prostate cancer risk (*BRCA1* and *BRCA2*) [36]. Most cases conserved the gene mutations between tissues and organoids (Figure 4c). To sum up, all results showed that the advanced prostate cancer organoids could mostly preserve the tumor heterogeneity of the matched tissues, based on pathological features and genomic profiles.

### 3.3. Drug Screening Applications of Advanced Prostate Cancer Organoids

**Figure 5:**
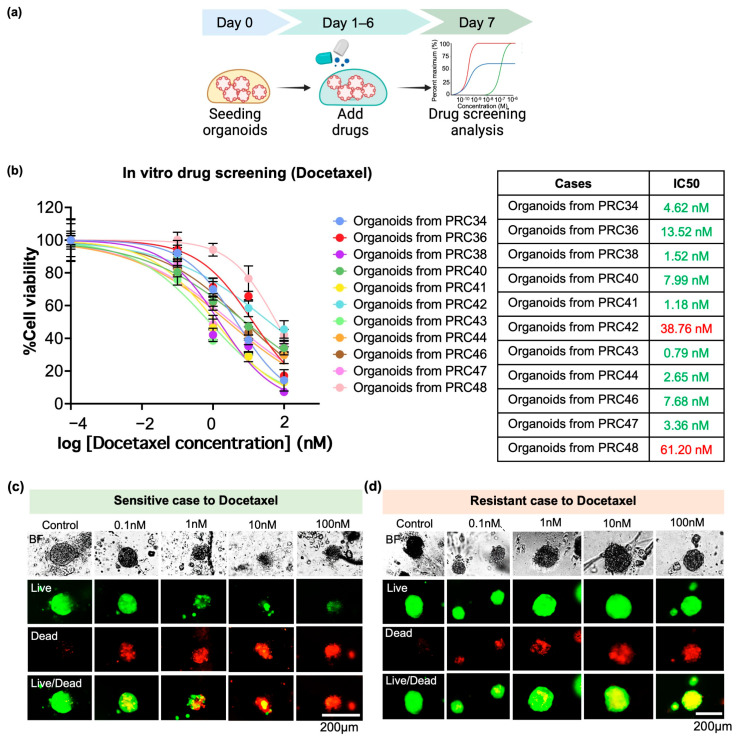
Drug sensitivities of developed organoids upon docetaxel chemotherapy. (**a**) Schematic image showing timeline of drug testing from seeding organoids (Day 0) to drug screening analysis (Day 7). (**b**) Percentage of cell viability measured by ATP-based luminescence assay (CellTiter-Glo), presented as a line graph (N = 11 cases; at least duplicate experiments per case). Half-maximal inhibitory concentration (IC50) values were calculated, and in vitro drug responses were interpreted using an IC50 cut-off of 20 nM. Green and red IC50 values indicate docetaxel-sensitive and docetaxel-resistant organoid responses, respectively. In addition, representative images of cases sensitive (**c**) and resistant (**d**) to docetaxel were demonstrated by live/dead fluorescence after 7 days of drug treatment.

To test if the developed organoids are feasible for screening drug responses with a short turnaround time, the organoids were screened using both chemo and hormone drug regimens (docetaxel and enzalutamide), based on clinical practice guidelines [2]. Docetaxel is a first-line agent which inhibits division of cancer cells and reduces expression of anti-apoptosis genes [37]. Enzalutamide is an androgen receptor inhibitor for hormone-sensitive prostate cancer treatment and is typically used to enhance the overall survival of patients [38]. A schematic timeline of the drug screening is shown in Figure 5a. Briefly, the organoids were seeded and re-grown for a day and then they were exposed to 0, 0.1, 1, 10, and 100 nM of docetaxel or 0, 0.01, 0.1, 1, and 10 µM of enzalutamide for 6 days. At the end of the treatment, the treated organoids were analyzed for drug sensitivity by quantification of ATP-based luminescent cell viability and qualification of fluorescence cell viability.

In our results, in vitro drug responses were obtained by half-maximal inhibitory concentration (IC50) values. Based on clinical outcomes of patients and previous studies, in vitro drug screening results were determined using the IC50 cut-off values of 20 nM for docetaxel and 10 µM for enzalutamide [14,39]. Overall, advanced prostate cancer organoids had IC50 values in the range of 0.79 to 61.20 nM for docetaxel and 0.07 to more than 10 uM for enzalutamide. The organoids were mostly sensitive to both drugs, although some cases were likely resistant to them. For instance, the organoids of the PRC43 case presented susceptibility to docetaxel, with an IC50 of 0.79 nM, but they did not respond to enzalutamide, with an IC50 greater than 10 µM. In another case, the organoids of PRC48 were resistant to docetaxel and less sensitive to enzalutamide, with IC50 values of 61.20 nM and 7.55 uM, respectively (Figure 5b and Figure S3b). These results corresponded to the qualification of Calcein AM (live) and BOBO-3 iodide (dead) staining. The sensitive cases exhibited decreased living cells in a dose-dependent manner (Figure 5c and Figure S3c). On the other hand, the resistant cases still exhibited expression of living cells, despite increasing drug concentrations (Figure 5d and Figure S3d). Therefore, these results demonstrated that the generated organoids were capable of predicting both chemo- and hormone therapies, resulting in drug recommendation within a month.

### 3.4. Correlation of In Vitro Drug Screening with Clinical Response

**Figure 6:**
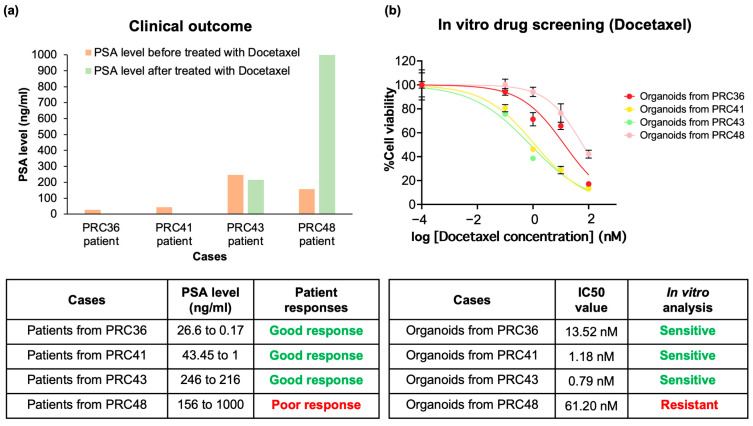
Correlation of in vitro drug screening (Docetaxel) with clinical response. (**a**) Bar graph reporting the prostate-specific antigen levels before and after docetaxel treatment for patients PRC36, PRC41, PRC43, and PRC48. (**b**) Percentage of cell viability in correlated cases compared with clinical levels. The bottom tables provide a summary of clinical outcomes correlated with in vitro results.

To evaluate whether the drug responses observed in organoids reflected patient treatment outcomes, we compared the docetaxel sensitivity of the PDOs with clinical responses measured by changes in prostate-specific antigen (PSA) levels. PSA is routinely used in clinical guidelines as a biomarker for monitoring treatment response in prostate cancer, where a decline indicates a reduction in the viable tumor burden and rising PSA suggests non-response [2]. Because PSA secretion is tightly regulated by luminal epithelial cell activity and decreases when viable prostate cancer cells are lost, we hypothesize that the biological mechanism underlying PSA decline in vivo mirrors the reduction in organoid viability observed in vitro [2,6,7]. All four patients (PRC36, PRC41, PRC43, and PRC48) presented with advanced prostate cancer (a Gleason score of 8–9) and were treated with androgen deprivation therapy (ADT) followed by docetaxel. According to the clinical data, patients PRC36, PRC41, and PRC43 demonstrated PSA reductions of 99.4%, 97.7%, and 12.2% after treatment, indicating biochemical response. These outcomes aligned with the in vitro drug screening results. For in vitro drug-response classification, we defined docetaxel sensitivity using IC50 values and adopted 20 nM as the cut-off, based on prior prostate cancer organoid studies demonstrating that concentrations below this threshold reliably induce significant viability reduction [14,39]. PDOs with an IC50 ≤ 20 nM were therefore classified as sensitive, whereas PDOs with an IC50 > 20 nM were classified as resistant. Consistent with the clinical outcomes, the PDOs from PRC36, PRC41, and PRC43 exhibited docetaxel sensitivity with IC50 values of 13.52 nM, 1.18 nM, and 0.79 nM, respectively. In contrast, patient PRC48 displayed a more than 6.4-fold increase in PSA following docetaxel therapy, consistent with clinical non-response. The matched PDO also showed resistance to docetaxel, with an IC50 of 61.20 nM (Figure 6a,b). To further quantify the association between in vivo and in vitro responses, we performed a Fisher’s exact test comparing clinical PSA-based outcomes (response vs. non-response) with PDO drug responses (sensitive vs. resistant). The analysis yielded a *p* value of 0.25. Although this is not statistically significant due to the very small sample size (n = 4), concordance across all samples was complete: each PDO classification matched the corresponding clinical outcome, resulting in 100% accuracy and 100% specificity in predicting docetaxel response in this pilot cohort (Figure 6 and Figure S4). However, the number of correlated cases remains limited, and additional matched patient–PDO pairs will be required to enhance statistical power and validate the robustness of this relationship. Nevertheless, these findings demonstrate that the developed organoids were able to recapitulate real patient outcomes and show strong potential for accurately predicting functional drug responses. Importantly, our results further indicate that short-term PDO expansion using a cost-effective FBS-based medium is sufficient to preserve clinically relevant drug-response phenotypes.

## 4. Discussion

Patient-derived organoids (PDOs) have emerged as powerful platforms for modeling tumor heterogeneity and predicting therapeutic responses [11,12,13]. However, the prostate cancer organoids that are established from TUR-P specimens are hampered by a low success rate [11,17]. This is because of the damage to some cell populations caused by electric wires during tissue collection [2]. In this study, we optimized tissue processing and culture conditions to substantially improve the establishment rate of advanced prostate cancer PDOs and to evaluate their potential for rapid drug-response prediction.

For the first success factor, our optimized protocol emphasized the critical role of tissue digestion and the controlled size of tissue fragments produced during sequential mechanical and enzymatic dissociation. We proposed establishing organoids directly from tissue fragments, rather than fully dissociated single cells, because partially digested fragments minimize culture adaptation time and more effectively support early organoid growth. Previous studies have shown that the generation of prostate cancer organoids or cell clusters typically involves highly variable enzyme concentrations and incubation periods, ranging from 30 min to 2 h depending on sample quality and digestion protocol conditions [11,15,40]. However, our findings demonstrated that a more standardized and milder digestion approach was sufficient to achieve high-quality organoid initiation. Specifically, we optimized the enzymatic step by fixing the collagenase type 2 concentration at 5 mg/mL and found that a digestion period of approximately 15 min provided the most effective condition, yielding organoid-initiating tissue fragments of about 100 μm. These fragments were adequate to support efficient and reproducible organoid formation. The robust growth and improved success rate observed in our study are consistent with earlier reports highlighting the advantages of using tissue fragments for prostate cancer organoid derivation [11,40,41]. This improved establishment efficiency is likely associated with the preservation of cell–cell and cell–stroma interactions and the retention of native components of the tumor microenvironment, as reflected in our histological analyses in Figure 2. The progression of prostate cancer organoids was reported to be significantly induced by their heterogeneous microenvironment [42,43], particularly the supportive role of stromal cells [11,44]. Moreover, our protocol clarified that the initial size of tissue fragments substantially affects the growth kinetics of organoids. We therefore selected fragments smaller than 100 μm to ensure optimal growth efficiency, as summarized in Figure 1e,f. This optimized initial size range of tissue fragments is also consistent with previous findings showing that fragments within this dimension facilitate adequate nutrient and oxygen perfusion into the core, promoting cell viability and organoid expansion [45].

Another major contribution of this work is the development of a simplified FBS-based organoid medium as a cost-effective alternative to niche factor-dependent formulations requiring recombinant R-spondin, Noggin, and EGF [11,13,15]. By substituting multiple recombinant growth factors with FBS, our protocol substantially reduces cost, simplifies medium preparation, and improves practicality for laboratories attempting to establish patient-derived prostate cancer organoids. Importantly, the optimized FBS-based medium supported organoid expansion while preserving the malignant epithelial phenotype observed in the original tumors, without overgrowth of benign or stromal cells, as confirmed through detailed H&E and IHC staining. STR profiling and ddPCR further confirmed that each PDO line preserved the molecular and genomic hallmarks of its matched tumor. Collectively, these data establish that the FBS-based formulation is suitable for short-term culture of advanced prostate cancer PDOs while retaining tumor specificity. These results collectively confirm that the FBS-based formulation reliably supports short-term culture of advanced prostate cancer PDOs while preserving tumor specificity and integrity. These findings are consistent with prior studies reporting that FBS-containing media can support short-term PDO generation from thyroid, skin, gastrointestinal, and renal cancers for applications such as high-throughput drug screening and immune profiling. These findings align with prior studies demonstrating that FBS-containing media can support short-term PDO generation from thyroid, skin, gastrointestinal, and renal cancers for drug screening and immune profiling applications [19,20,46].

In terms of potential molecular mechanisms underlying these observations, fetal bovine serum (FBS) contains a diverse array of bioactive molecules capable of functionally substituting for canonical niche factors. These include insulin-like growth factors (IGF-1 and IGF-2) and insulin, which activate the PI3K/AKT and Wnt/β-catenin signaling pathways to maintain stemness and promote epithelial proliferation [47,48,49]. FBS also contains physiologic levels of testosterone—10% FBS typically yields ~0.1 ng/mL testosterone in culture medium—which is sufficient to enhance proliferation of AR-positive prostate cancer cells and support AR-dependent tumor growth [14,48]. Moreover, FBS provides cytokines and peptide mediators that activate AR-independent proliferative pathways, enabling the survival of heterogeneous tumor subpopulations frequently observed in advanced prostate cancer. Notably, FBS has been shown to induce IL-8 secretion [50,51], a cytokine strongly associated with inflammation-driven tumorigenesis, stemness maintenance, and prostate cancer progression [52]. Collectively, these biological properties offer a strong mechanistic rationale for how FBS can functionally replace multiple recombinant niche supplements while still sustaining the short-term expansion of advanced prostate cancer organoids.

While our study demonstrates that this simplified FBS-based formulation is a practical, accessible, and effective medium for short-term prostate cancer PDO establishment and functional drug testing, further optimization is necessary to advance its translational utility. Future work should include direct comparison between the FBS-based formulation and standard niche factor media, comprehensive profiling of FBS components using proteomic and metabolomic approaches, and batch-to-batch consistency assessments to ensure reproducibility. Mechanistic studies employing pathway perturbation will also be essential to elucidate how FBS-derived signals promote organoid proliferation and lineage maintenance.

To determine whether the advanced prostate cancer organoids generated in this study could serve as reliable tools for personalized medicine, we comprehensively characterized their cellular and genetic fidelity to the original tumors. Histopathological assessment showed that PDOs retained an epithelial morphology and malignant features comparable to their matched tissues. The organoid–tissue pairs also preserved key immunophenotypic markers, including CK5/6, CK8, and AR. As expected for advanced prostate cancer, most PDOs demonstrated strong CK8 expression and reduced CK5/6 expression, consistent with a luminal-dominant phenotype. AR expression was also largely preserved, in line with its reported sensitivity (94.8%) and specificity of about 81.4% for prostatic adenocarcinoma [53,54,55,56].

For genetic profiling analysis, the similarity of the organoid–tissue pairs was initially confirmed by performing STR analysis. These results displayed that the organoids could preserve short tandem repeat profiles similar to those of the original tissues. For instance, one set of organoids could capture type 2 tri-allelic patterns relating to the original tissue. In addition, we also validated the organoid–tissue pairs by ddPCR for characterizing CNVs of the *BRCA1* gene. Prostate cancer patients who had *BRCA1* pathogenic variant carriers were suggested to be at higher risk for aggressive prostate cancer [32,57,58]. The results showed that the organoids and tissues could recapitulate together with the loss of CNVs of the *BRCA1* gene. This indicated that the organoid–tissue pairs had the possibility of gene deletion.

To clarify that the organoids could preserve gene mutations that resembled mutations in the original tissues, the organoid–tissue pairs were validated by whole-exome sequencing. The results confirmed that the developed organoids could extensively recapitulate the gene mutation profiles of original tissues for SNVs and Indels with, on average, more than 70% similarity, corresponding to the reports of previous studies [13,59]. In addition, the organoids could also maintain mutations in prostate cancer-associated genes with high mutation frequencies [13,31,32], similar to the original tissues. We observed recurrent missense mutations in *FOXA1*, *TP53*, and *APC*, which were consistently preserved in all tissue–organoid pairs. This corresponded with previous studies that found that *FOXA1* and *TP53* alterations were the most prevalent alterations in advanced prostate cancer [32,60]. Evidently, *TP53* mutation has been widely associated with multiple cancer types [61] and strongly correlated with a high risk of developing aggressive prostate cancer [62,63]. Likewise, *APC* gene aberrations are commonly detected in prostate adenocarcinoma and have been reported to confer a significantly elevated risk of disease aggressiveness [64,65].

Furthermore, we found that the tissues and organoids in all cases carried variants in DNA repair-associated genes, especially *BRCA1*. This corresponded to the CNV profiles of the *BRCA1* gene determined by ddPCR, which showed abnormality of the gene in all organoid and tissue pairs. For AR mutation, this gene was not only significantly enriched following prostate cancer progression [66], but was also associated with prognosis of time to progression [35]. Typically, the alterations of the AR gene in castration-sensitive prostate cancer were less dominant than in the resistant stage [32,66]. Consistent with this, no *AR* mutations were detected in the PRC40 and PRC43 organoid cases derived from castration-sensitive prostate cancer patients. On the other hand, PRC48 cases, derived from castration-resistant prostate cancer patients, exhibited a missense mutation in the tissue sample but not in the corresponding organoid. This discrepancy may be attributed to tumor mosaicism, reflecting spatial heterogeneity within different regions of the same cancer tissue [67]. Additionally, it could be related to the limitations of whole-exome sequencing (WES) with a low read depth, which may be insufficient to detect structural variants or low-frequency mutations within the *AR* gene [65,68]. Overall, these results indicated that the generated organoids efficiently imitated the tumor origins at both cellular and genetic levels.

Importantly, we evaluated whether advanced prostate cancer PDOs could function as predictive tools for patient-specific therapeutic responses. Drug screening was performed in parallel with clinical treatment, and most PDOs demonstrated sensitivity to docetaxel. In particular, PDOs from PRC36, PRC41, and PRC43 showed strong docetaxel sensitivity, which corresponded with substantial reductions in PSA levels observed in the patients after treatment. In contrast, the PDO derived from PRC48 exhibited marked resistance to docetaxel, consistent with the patient’s significant PSA increase following therapy (Figure 5 and Figure 6). PSA is the clinical gold-standard biomarker for monitoring treatment response in advanced prostate cancer [2], and its decline reflects a reduction in viable luminal tumor epithelial cells. Because PSA production is directly linked to the number and functional activity of living tumor cells, the decrease in PSA observed in vivo is biologically consistent with the reduction in PDO viability measured in vitro. Regarding these correlations, all four matched patient–PDO pairs exhibited concordant outcomes, corresponding to approximately 100% specificity and accuracy in this pilot analysis. However, to improve this study, the number of samples derived from a wide variety of prostate cancer patients should be increased to enhance the statistical power of drug-response predictions (Supplementary Figure S4).

To summarize, the advanced prostate cancer organoids generated in this study demonstrated strong potential as functional models for predicting patient responses and supporting clinical decision-making within a short turnaround time. Although our study proved the capacity of the organoids for precision drug screening, several limitations remain. First, the mechanism by which the FBS-based culture medium promotes organoid proliferation is not yet fully understood. Elucidating the molecular pathways and bioactive components of FBS, through proteomic profiling and mechanistic analyses, will be important for optimizing this medium and potentially extending its applicability to organoid models from other cancer types. Second, although the establishment success rate in this study (62.07%) represents a marked improvement over previous reports, it remains insufficient for routine clinical implementation. Factors contributing to unsuccessful PDO generation likely include prolonged ischemia time before tissue processing, microbial contamination, and low epithelial cellularity, particularly in necrotic or fibrotic tumors. Future workflow refinements, including rapid tissue transfer, improved contamination control, and epithelial enrichment strategies, may enhance success rates. Third, while *BRCA1* copy number loss was consistently maintained between tissues and matched organoids, ddPCR analysis indicated heterozygous rather than homozygous deletion in all cases. Finally, the relatively small sample size limits the statistical power and generalizability; expanding the number and diversity of patient samples in future clinical studies will be essential for validating predictive accuracy and establishing robust success criteria. Collectively, these improvements will be critical for translating TUR-P-derived PDOs into a reliable, scalable, and clinically actionable platform for personalized therapeutic decision-making.

## 5. Conclusions

In this study, we established an improved culture protocol that significantly enhances the success rate of deriving advanced prostate cancer organoids from TUR-P specimens. The resulting organoids faithfully recapitulated the cellular and genetic heterogeneity of their matched tumors. In this pilot cohort, the organoids also showed encouraging concordance with patient drug responses, suggesting that TUR-P-derived PDOs may serve as a promising functional screening model with the potential to estimate patient-specific treatment responses within a month after surgery. Further validation in larger, clinically diverse cohorts will be essential to confirm their predictive value and support future clinical translation.

## Figures and Tables

**Table 1:** Characterization of advanced prostate cancer patients enrolled in organoid development.

Age	Years
Median (range)	71.34 (40–90)
Types of tissue collections	Cases
Transurethral Resection of the Prostate	28
Radical prostatectomy	1
Gleason score	N (%)
6	1 (3.45%)
7	6 (20.69%)
8	7 (24.14%)
9	13 (44.83%)
10	2 (6.90%)
PSA level before treatments	ng/mL
Median (range)	236.97 (1–1561)
PSA level after treatments	ng/mL
Median (range) of 26 sensitive patients	26.10 (0.025–300)
Median (range) of 1 resistant patient	>1000

## Data Availability

All data generated or analyzed during this study are contained within the article and its Supplementary Materials.

## Supplementary Materials

The following supporting information can be downloaded at https://www.mdpi.com/article/10.3390/cancers17243973/s1, Figure S1: The passaging capacity of prostate cancer organoids; Figure S2: Histological and immunohistological analyses of generated organoids compared to the matched tumors; Figure S3: Drug sensitivities of developed organoids upon enzalutamide anti-androgen drug; Figure S4: Correlation between in vitro drug screening results of organoids and patient treatment responses determined using Fisher’s exact test.
